# Pain in Down's Syndrome

**DOI:** 10.1100/tsw.2006.27

**Published:** 2006-01-26

**Authors:** Federica Mafrica, Daniela Schifilliti, Vincenzo Fodale

**Affiliations:** Department of Neuroscience, Psychiatric and Anesthesiological Sciences, University of Messina, School of Medicine, Policlinico Universitario “G. Martino”, 98125 Messina, Italy

**Keywords:** analgesia, anesthesia, Down's syndrome or trisomy 21, brain, pain, pain measurement, pain treatment

## Abstract

Pain is a homeostatic mechanism that intervenes to protect the organism from harmful stimuli that could damage its integrity. It is made up of two components: the sensory-discriminative component, which identifies the provenance and characteristics of the type of pain; and the affective-motivational component, on which emotional reflexes, following the painful sensation, depend.There is a system for pain control at an encephalic and spinal level, principally made up of the periaqueductal grey matter, the periventricular area, the nucleus raphe magnus, and the pain-inhibition complex situated in the posterior horns of the spinal cord. Through the activation of these pain-control systems, the nervous system suppresses the afference of pain signals. Endogenous opioids represent another analgesic system.In the course of various studies on pain transmission in Down patients, the reduced tolerance of pain and the incapacity to give a qualitative and quantitative description emerged in a powerful way. All of these aspects cause difficulty in evaluating pain. This is linked to several learning difficulties. However, it cannot be excluded that in these anomalies of pain perception, both the anatomical and the neurotransmitter alteration, typical of this syndrome, may hold a certain importance.This fact may have important clinical repercussions that could affect the choice of therapeutic and rehabilitative schemes for treatment of pathologies in which pain is the dominant symptom, such as postoperative pain. It could influence research on analgesics that are more suitable for these patients, the evaluation of the depth of analgesia during surgical operation, and ultimately, absence of obvious pain manifestations. In conclusion, alterations of the central nervous system, neurotransmitters, pain transmission, and all related problems should be considered in the management of pain in patients with Down's syndrome, especially by algologists and anesthesiologists.

## INTRODUCTION

Pain is a universal human experience. Usually, pain is a normal homeostatic mechanism that forces an organism to avoid or reduce injury. The body has many pain messengers, receptors, and neural pathways, all employed in intercepting the pain sensation. The perception of a stimulus that leads to, or has the potential to cause, tissue injury is termed "nociception". In clinical disease states, pain may be the result of tissue damage or aberrant signal processing[1].

Pain has two components. The first is the sensory-discriminative component and provides information on location, modality, and intensity of stimuli. The second is the affective-motivational component and refers to the emotional responses (fear, distress, etc.) and to the urge to respond that is evoked by the somatic sensation, at a cortical level; these two components appear to be located in different regions[2].

While the emotional experience is thought to be represented in the right hemisphere, the left hemisphere and adjacent dorsal insula have a dominant role in the early sensory-discriminative dimension processing[3]. Anatomic and electrophysiologic data show that these cortical regions receive direct nociceptive thalamic input. The anterior cingulate cortex is closely related to unpleasantness caused by pain and may subserve the integration of general affect, cognition, and response selection[4].

## NEUROANATOMY AND NEUROPHYSIOLOGY OF PAIN

Several cortical areas have been shown to be involved in the processing of painful stimuli: primary somatosensory cortex, secondary somatosensory cortex and its vicinity to the parietal operculum, insula, anterior cingulate cortex, and prefrontal cortex. These areas probably process different aspects of pain in parallel. Previous psychophysical research has emphasized the importance of separating the pain experience into sensory-discriminative and affective-motivational components[5].

Peripheral nociceptive messages are conveyed by a mosaic of unmyelinated free fibers distributed throughout cutaneous, muscular, and articular tissues, and within the visceral walls. They are then transmitted via various nerve endings (polymodal nociceptors) by small-diameter A delta and C fibers. It is nevertheless difficult to ascertain whether these small-diameter fibers are involved only in nociception (specific nociceptors) or if pain causes an excessive activation of these receptors, which, under normal conditions, play a role in the reflex that regulates various functions (nonspecific nociceptors)[6].

Nociceptors can be classified according to their responses to mechanical, thermal, and chemical stimuli (skin receptors, nociceptors of deep tissues, and visceral, free nerve endings)[7]. Their essential functions are to transduce noxious stimuli, which activate them, into depolarizations that trigger action potentials, which are then conducted from the peripheral sensory site to the synapse in the central nervous system, and converted into neurotransmitter release at the presynaptic terminations depending on ion channels[8].

The different nociceptor types are probably characterized by different sets of receptor molecules in the membrane of the nociceptive endings. Nociceptive cells are present in the superficial laminae and neck of the dorsal horn. At a spinal level, neuroplastic-sensitizing processes — that are assumed to underlie the allodynia and hyperalgesia of pain patients — take place[9].

The presence of substance P in demyelinated sensory fibers and in the small- and medium-size neurons of spinal dorsal horn substantia gelatinosa, gives a structural basis for the hypothesis that substance P plays an important role as a mediator in the processing of nociceptive information[10].

Spinal lamina I neurons, expressing the substance P receptor, have been shown to play a role in the transmission of somatic, inflammatory, and neuropathic pain. Acute noxious visceral stimuli induce activation of spinal lamina I neurons expressing the substance P receptor. After visceral inflammation, there is a marked increase in both the number and rostrocaudal extent of lamina I substance P receptor neurons activated in response to both a normal non-noxious and noxious distention of the colon[11].

Nociception is related to the mechanisms elicited by the stimuli that threaten the integrity of the organism. At a peripheral level, unmyelinated C fibers (C polymodal nociceptors) or fine myelinated A delta fibers are excited by noxious stimulation, and, directly or indirectly, by inflammatory processes. Nociceptive afferent fibers terminate in the superficial laminae of the dorsal horn of the spinal cord where information is integrated and controlled[12].

The first synapses are modulated by excitatory amino acids and many peptides, at the end of the peripheral nerves in the dorsal horn of the spinal cord. Many supraspinal sites are activated: the brain stem, the pontomesencephalic regions, the thalamic sites, and the cortex[13].

## PAIN-CONTROL SYSTEM

The periaqueductal grey matter and the nucleus raphe magnus and adjacent structures of the rostral ventromedial medulla, with their projections to the spinal dorsal horn, constitute the "efferent channel" of a pain-control system that "descends" from the brain onto the spinal cord. This evidence is reviewed and organized to support the idea that persistent nociception simultaneously triggers descending facilitation and inhibition[14].

The neural basis for this antinociceptive action is reasonably well understood, with strong evidence that activation of a class of rostral ventromedial medulla neurons termed *off-cells* exerts an inhibitory effect on nociception. However, it has recently become clear that this system can facilitate nociception through the activation of a class of neurons termed *on-cells* as a mediating descending facilitation[15].

The importance of the nucleus raphe magnus in descending inhibitory control of nociception is clear, but it is not known whether these effects are equivalent in different types of nociception[16]. Primary fibers travel from the periphery to the dorsal horn where they synapse on secondary neurons and interneurons. When activated, interneurons exert inhibitory influences on further pain signal trafficking[17].

Descending systems also modulate pain sensitivity at a spinal level. The descending 5-hydroxytryptamine (5-HT) system has a tonic inhibitory function, with diurnal fluctuations in intensity. The ascending 5-HT system may influence the results of some complex tests for pain sensitivity[18]. Serotonin (5-HT) is present in central and peripheral serotoninergic neurons, is released from platelets and mast cells after tissue injury, and exerts algesic and analgesic effects depending on the site of action and the receptor subtype[19].

Spinal 5-HT-3 receptors are involved in the modulation of pain sensitivity; activation of these receptors inhibits nociceptive reactions, while blockade of 5-HT-3 receptors potentiates the nociceptive response via modulation of excitability of gamma-aminobutyric acid (GABA) ergic interneurons[19]. The 5-HT-3 receptor antagonists, via a central and/or peripheral action, have been shown to reduce secretion and motility in the gut and to possess clinical utility in irritable bowel syndrome, and possibly in other visceral pain disorders[20]. In the central nervous system, the 5-HT-3 receptors have been localized in the area postrema, nucleus tractus solitari, nucleus caudatus, nucleus accumbens, amygdala, hippocampus, entorhinal, frontal, cingulate cortex, and in the dorsal horn ganglia. Moreover, 5-HT-3 receptors modulate the release of neurotransmitters and neuropeptides like dopamine, cholecystokinin, acetylcholine, GABA, substance P, and serotonin itself[21].

Also, the muscarinic acetylcholine receptors have been widely reported as pharmacological targets for the treatment of pain[22]. The spinal cholinergic system and muscarinic receptors are important for regulation of nociception. Activation of spinal muscarinic receptors produces analgesia and inhibits dorsal horn neuronease through potentiation of GABA release. Spontaneous inhibitory postsynaptic currents are recorded in lamina II neurons using whole-cell voltage-clamp recordings in rat's spinal cord slices[23].

Until recently, few GABA acting drugs were available and were prescribed mostly for relieving muscle spasms, anxiety, and epilepsy, but rarely for pain. The basic metabolic pathway of GABA is well known and research is now beginning to understand the function of this neurotransmitter in the complex circuitry underlying pain, especially in the context of nerve injury[24].

Nociceptive afferents have their neurons in the dorsal root ganglion and contact second-order neurons in the dorsal horn or, less often, in the medulla. Modulation of the pain signal in the dorsal horn involves local inhibitory and facilitatory interneurons as well as diverse excitatory and inhibitory neurotransmitters. This central sensitization is thought to be mediated largely through the *N*-methyl-D-aspartate (NMDA) receptor complex[25].

The role of NMDA mechanisms in spinal pathways mediating acute nociceptive input to the somatosensory cortex is not clear. In a study, the effect of NMDA antagonists on nociceptive C fiber transmission to the primary somatosensory cortex (SI) was investigated. It was concluded that spinal NMDA receptor mechanisms amplify the acute transmission of nociceptive C fiber input to primary somatosensory cortex in a frequency-dependent way[26].

The nervous and immune systems communicate with each other by use of cytokines and neuropeptides. The activation of opioid production and release from immune cells may represent a new approach to the development of peripherally acting analgesics. Because such drugs would be targeted towards events in peripheral injured tissue, these analgesics should lack unwanted central side effects typically associated with opioids[27].

A current concept proposes that during inflammatory processes, endogenous opioid peptides may be secreted from immunocytes, occupy peripheral opioid receptors on sensory nerve endings, and produce analgesia by inhibiting the excitability of these nerves or the release of proinflammatory neuropeptides[28]. In fact, during inflammatory processes, opioid receptors are transported from dorsal root ganglia towards the peripheral sensory nerve endings. At the same time, immune cells containing endogenous opioid peptides accumulate within the inflamed tissue. Environmental stimuli (e.g., stress) as well as releasing agents (e.g., corticotrophin-releasing factor, cytokines) can set free these opioid peptides to interact with the neuronal opioid receptors and elicit local analgesia[29].

Recent studies have revealed that cholecystokinin is up-regulated in the rostral ventromedial medulla during persistent opioid exposure. Cholecystokinin is both antiopioid and pronociceptive, and activates descending pain facilitation mechanisms from the rostral ventromedial medulla, enhancing nociceptive transmission at the spinal cord and promoting hyperalgesia[30].

The analgesic actions of opioids can be modified by endogenous "antiopioid" peptides, including cholecystokinin. Cholecystokinin is now thought to have a broader, pronociceptive role, and to contribute to hyperalgesia in inflammatory and neuropathic pain states. The selective activation of rostral ventromedial medulla on-cells by cholecystokinin is sufficient to produce thermal hyperalgesia and indicate that the antiopioid and pronociceptive effects of this peptide are mediated by actions on different rostral ventromedial medulla cell classes[31].

## PAIN EXPERIENCE IN DOWN'S SYNDROME

Pain is subjective and multidimensional. Responses to pain are expressed by self-port, changes in behavior, and changes in physiological indices. Several self-port methods, observation scales, and physiological measures have been developed and used to assess pain. The subjective dimension of pain hampers its measurement. People who are profoundly cognitively impaired are not able to express their pain verbally[32].

Individuals with Down's syndrome do not always exhibit signs of distress in reaction to noxious stimuli, if compared to the general population. A pilot study was designed to measure the ability of individuals with Down's syndrome to detect and express sensation in comparison with healthy volunteers. The results suggested the individuals with Down's syndrome are not insensitive to pain, however, they do express pain or discomfort more slowly and less precisely than the general population[33].

Another study explored several methods for assessing pain during venipuncture in children, using classic and modified scales (Visual Analog Scale, Eland Scale, Faces Scale, Cube Test, Modified Eland Scale, and Modified Faces Scale), to evaluate the children's response to simplified tools. The Eland Scale proved difficult to use, especially for Down's syndrome children, while its modified version was easier. The patients showed a limited capacity for verbal and behavioral expression in reaction to the painful stimulus (especially the Down cases)[34].

An experimental protocol was developed with which individuals with mild moderate/severe learning difficulties were trained (unspecified moderate/severe learning difficulties and Down's syndrome) in the use of a heat-pain threshold measurement on the hand. The measurement was performed using both the method of limits, which relies on reaction time, and the method of levels, which is reaction-time free. From this experiment, it would appear that individuals with moderate/severe learning difficulties are not only pain sensitive, but also more sensitive to heat-pain than normal[35].

To investigate the ability of parents to perceive pain experienced by their offspring (204 children with Down's syndrome and 161 siblings without the syndrome), data were gathered using the Oral Assessment in Down's syndrome Questionnaire in a cross-sectional survey designed in France. Parental reports of difficulty discerning if their child with Down's syndrome was in pain did not change with age of the child, remaining prevalently in the 28–32% range. Reports of difficulty in discerning where children felt pain diminished with age from 74–27%. Parental perception of pain is less discriminating for children with Down's syndrome than for their offspring without Down's syndrome[36].

Individuals with Down's syndrome have a decreased perception of pain, which could be related to raised opioid peptides in the frontal cortex of these people. Increased concentration of the analgesic opioids leuenkephalin and dynorphin in an area of the brain involved with integrating physiological responses to environmental stimuli could explain the increased pain threshold in Down's syndrome patients[37].

Differences regarding endogenous opioids in the postmortem frontal cortex of adult patients with Down's syndrome, of patients with Alzheimer's disease, and of neurologically healthy people were investigated using specific radioimmunoassay. Results have demonstrated that there is an increase in the levels of leuenkephalin and dynorphin A in the frontal cortex of patients with Down's syndrome as compared to the control group[38]. Several animal models of Down's syndrome with triplication of genes from the Down's syndrome critical region were generated, including mouse trisomy 16 (Ts16) and a partial trisomic mouse, Ts65Dn[39], and studies on pain transmission in Down's syndrome was carried out. Since pain transmission and expression may be limited in people with mental disabilities, including Down syndrome, responsiveness to nociception in Ts65Dn mice was compared with that in their control littermates. The results indicate that trisomic mice present an overall depressed responsiveness to nociceptive stimulation[40].

In addition, the subnormal peripheral somatosensory function might include the transmission of painful stimuli that come, along with the other dysfunctions of this syndrome, from the acquisition of trisomy 21[41]. A study that considered demographic data, medical backgrounds, and physical and functional tests, compared older people with Down's syndrome and a control group with regard to clinical characteristic, functional status, and sensorimotor functions. During this study, it was postulated that their slower responses may be explained by a less physically active lifestyle that may accelerate the onset of disease, resulting in symptoms associated with aging that are detrimental to health[42].

Middle-latency somatosensory-evoked potentials (MLSEPs) were recorded from 19 scalp electrodes in 19 patients with Down's syndrome, in 13 age-matched normal controls, and in 11 normal-aged individuals. MLSEPs of Down's syndrome subjects show a peculiar increase in amplitude that could be supported by particular neurometabolic and/or neuropathologic changes[43].

Sensory neurography of the median nerve was performed bilaterally on six children with Down's syndrome, aged 11–16 years, and on ten healthy controls of the same age. The sensory nerve conduction velocities from the thumbs and third fingers were recorded and were significantly subnormal in the Down group. Sensory nerve action potentials were also lower in the Down children and the difference was particularly evident following stimuli of thumbs. This suggests that an impaired peripheral somatosensory function should be added to previously known symptoms constituting Down's syndrome[44].

Using a standardized neuropsychological test, tactual finger discrimination, graphaesthesia, and stereognosis were studied in a group of 11 children aged 7–11 years with Down's syndrome. The results suggest that tactual perception is subnormal in children with Down's syndrome[45].

To investigate the sensory functions that go from the peripheral towards the central pathways in infants with Down's syndrome, sensory-evoked potentials were used; 55 infants, 30 with Down's syndrome and 25 controls, were examined by the use of multimodal-evoked potentials and short-latency–evoked potentials. The results indicate that various sensory deficits occur in patients with Down's syndrome during their first year of life[46].

## CONCLUSIONS

The complex qualitative and quantitative evaluation of pain in patients with Down's syndrome is linked to moderate/severe learning difficulties typical of this syndrome, whose predominating deficit concerns abstract thinking and spatial orientation. Therefore, Down subjects are unable, either verbally or through signs, to describe the features of pain sensation. These observations allow attention to be focused on the impossibility, or high difficulty, encountered in pain measurement in these patients.

The anatomical and neurotransmitting alterations, such as those of the cholinergic and the serotoninergic systems, could play a part in nociception and in the pain-control system of these patients. This fact may have important clinical repercussions that could affect, for example, the choice of therapeutic and rehabilitative schemes for treatment of pathologies in which pain is the dominant symptom, in the treatment of postoperative pain, in research on analgesics that are more suitable for these patients, and in the evaluation of the depth of analgesia during surgical operation, even in the absence of obvious pain manifestations. The latter could be overcome by using an adequate monitoring technique during surgery.

Moreover, other alterations that involve neurotransmission in subjects with Down's syndrome include the cholinergic system, which shows an important decrease, the GABA system, the noradrenergic system, and glutamate transmission. For example, considering that Down's syndrome exhibits cholinergic deficits, the cell lines derived from trisomy 16 mice have been further examined with reference to the cholinergic function of brain and spinal cord tissue compared with that of age-matched controls. The results indicate that the TS16 condition in mice significantly modified the cholinergic function in the brain and, to a lesser degree, in the spinal cord, suggesting that the higher gene dosage inherent in the trisomic condition affects cholinergic neurons in different regions of the central nervous system in a differential fashion[47]. Moreover disturbance of the central serotoninergic system has been suggested in Down's syndrome. In this syndrome, the 5-HT concentration in blood platelets is below normal, but the mechanism behind this is controversial. Recently, evidence has accumulated indicating a decreased active transport of 5-HT, possibly due to lowered activity of $Mg^{++}$-dependent $Na^{+}$-$K^{+}$-stimulated adenosine triphosphatase[48].

In conclusion, the alteration of the central nervous system, neurotransmitters, pain transmission, and all related problems should be considered in the management of pain in patients with Down's syndrome, especially by algologists and anesthesiologists.

## ACKNOWLEDGMENTS

Financial support used for the study was provided solely from departmental sources.
